# Pollution caused by nanoplastics: adverse effects and mechanisms of interaction *via* molecular simulation

**DOI:** 10.7717/peerj.13618

**Published:** 2022-07-25

**Authors:** Yamara Matos Oliveira, Nathalia Salles Vernin, Daniele Maia Bila, Marcia Marques, Frederico Wanderley Tavares

**Affiliations:** 1 Chemical Engineering Program, Alberto Luiz Coimbra Institute for Graduate Studies and Research in Engineering (COPPE), Federal University of Rio de Janeiro, Rio de Janeiro, RJ, Brazil; 2 Department of Sanitary and Environmental Engineering, Rio de Janeiro State University, Rio de Janeiro, RJ, Brazil; 3 School of Chemistry, Federal University of Rio de Janeiro, Rio de Janeiro, RJ, Brazil

**Keywords:** Microplastics, Molecular simulation, Nanoplastics, Adsorption, Adverse effects

## Abstract

The continuous increase in the production of synthetic plastics for decades and the inadequate disposal of plastic waste have resulted in a considerable increase of these materials in aquatic environments, which has developed into a major environmental concern. In addition to conventional parameters, the relevance of the environmental monitoring of microplastics (MPs) and nanoplastics (NPs) has been highlighted by the scientific community due to the potential adverse effects these materials pose to the ecosystem as well as to human health. The literature has registered an increasing interest in understanding the mechanisms, at the molecular level, of the interaction between NPs and other compounds using molecular simulation techniques. The present review aims to: (i) summarize the force fields conventionally used to describe NPs by molecular simulations; (ii) discuss the effects of NPs in the structural and dynamical properties of biological membranes; (iii) evaluate how NPs affect the folding of proteins; (iv) discuss the mechanisms by which NPs adsorb contaminants from the environment. NPs can affect the secondary structure of proteins and change the lateral organization and diffusion of lipid membranes. As a result, they may alter the lipid digestion in the gastrointestinal system representing a risk to the assimilation of the nutrients by humans. The adsorption of contaminants on MPs and NPs can potentiate their harmful effects on human health, due to a possible synergism. Therefore, understanding the mechanisms involved in these interactions is crucial to predict dangerous combinations and outline action strategies that reduce negative impacts on ecosystems and human health. Depending on the chemical properties of contaminants and NPs, electrostatic and/or van der Waals interactions can be more relevant in explaining the adsorption process. Finally, we conclude by highlighting gaps in the literature and the critical aspects for future investigations.

## Introduction

Plastic materials are decisive in several aspects of human life because their physical and chemical properties result in high durability and strength, low production cost, and weight. Nonetheless, its high durability is not without negative effects; even with its great potential to be recycled, tons of plastics become polluting agents in the environment in the course of time. In 2019, approximately 368 million tons of plastics were produced worldwide (Plastics Europe, 2020), but only 6% to 26% were recycled (Alimi et al., 2018; Li et al., 2021a) and around 10% eventually ended up in the ocean (Collard et al., 2017; Wang et al., 2019a). As a result of these plastics being exposed to chemical, physical and biological agents, they begin to degrade into microplastics (size < 5 mm) and nanoplastics (size < 100 nm) (Toussaint et al., 2019). Moreover, some plastics have already been designed to be microplastics (MPs).

The COVID-19 pandemic has been an aggravating factor in this regard, and, since its beginning, the use of disposable plastics in the health sector has greatly increased. A recent study estimated that in 2020, 1.56 million masks might have ended up in the ocean (Phelps Bondaroff & Cooke, 2020; Peng et al., 2021). Among all the plastic disposals related to COVID-19, it was estimated that, from the beginning of the pandemic until August 2021, a total of approximately 25.9 tons had reached the oceans, including 12.3 tons of micro and nanoplastics (Peng et al., 2021).

**Figure 1:**
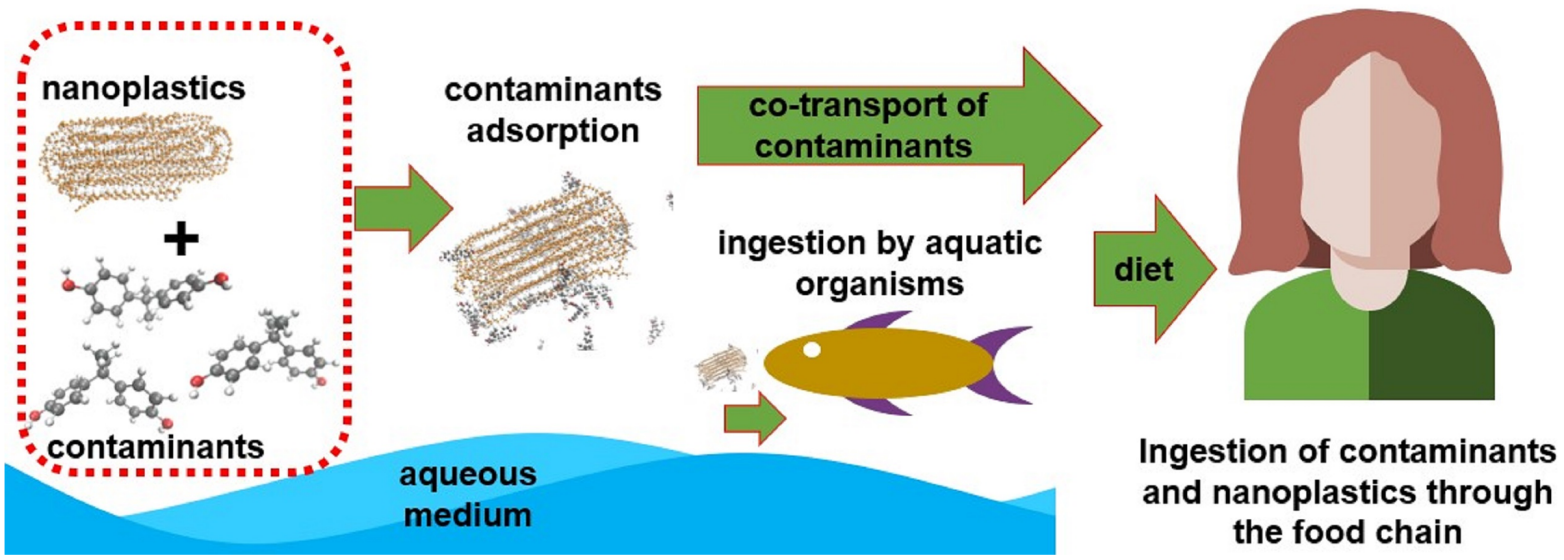
A possible transport scheme of pollutants adsorbed on NPs through the food chain.

Several studies prove the bioaccumulation of these particles in living marine life, indicating a direct route for contact with humans through the food chain as shown in the literature review conducted by Toussaint et al. (2019). Other access routes would be ingestion or contact with contaminated water, exposure to aerosols containing nanoplastics (NPs) and contaminated air (associated mainly with textile industry), as well as contact with skincare products and cleaning products that have NPs in their composition (Hüffer et al., 2017; Lehner et al., 2019). It is already known that these NPs and MPs have a high surface/volume ratio and, consequently, great tendency to adsorb other substances. It is important to highlight that, depending on the polymer’s glass transition temperature, the absorption of compounds can also occur. So, in addition to the presence of NPs and MPs, it is necessary to know how to deal with other types of pollutants adsorbed on its surface (Li et al., 2021b). Many experimental studies which have been conducted in recent years proved the sorption of antibiotics (Guo, Liu & Wang, 2019; Chen et al., 2021; Li, Zhang & Zhang, 2018), heavy metals (Guo, Liu & Wang, 2020; Almeida, Manjate & Ramos, 2020), pesticides (Li et al., 2021a), polycyclic aromatic hydrocarbons (PAH) (Tan et al., 2019; Sørensen et al., 2020), and other organic pollutants (Wang & Wang, 2018) in NP; these studies also showed how NPs may act as transport vehicles for these contaminants. Figure 1 represents a possible transport scheme of pollutants adsorbed on NPs through the food chain.

MPs and NPs with or without adsorbed pollutants, cause adverse effects to several species. Reichert et al. (2018), Okubo, Takahashi & Nakano (2018) and Okubo, Tamura-Nakano & Watanabe (2020) demonstrated the negative effects in different corals when exposed to MPs. Some studies show that the combined exposure of MPs/NPs with adsorbed metals can heighten toxic effects in aquatic test organisms (Lee et al., 2019; Qiao et al., 2019). Similar evidence were observed in other experimental studies carried out with organic pollutants such as phthalates, PAH, pharmaceuticals, and flame retardants accumulated on the MP surface (Li et al., 2020; Nobre et al., 2020; Pittura et al., 2018). It is important to highlight that most experiments are carried out under laboratory conditions and employ high concentrations of MPs/NPs and pollutants, which are unrealistic conditions when compared to aquatic environments. Few studies indicate that the presence of pollutants adhered to the surface of MPs can lead to synergistic, antagonistic, and additive effects under real conditions (Bhagat, Nishimura & Shimada, 2021; Rodrigues et al., 2019). Regarding human exposure to MPs and NPs, there is a great concern in determining, first, effectively how sizeable the contamination is, and, second, the main contact mechanisms (Lehner et al., 2019; Wright & Kelly, 2017). However, the possible effects on human health tend to be estimated, as studies that prove the problems with exposure to nanoplastics have been materializing (Gopinath et al., 2019; Domenech et al., 2021).

The use of molecular simulation techniques, such as molecular dynamics and Monte Carlo simulations, is an alternative strategy to understand the possible health harms due to human exposure to NPs (Hollóczki, 2021). Molecular simulation is a powerful tool for studying phenomena at the nanometric scale and has been remarkably successful in predicting macroscopic thermodynamic and dynamic observables for various systems. It is continually growing as an option for describing system properties under conditions that experimental determinations are difficult to acquire (Tuckerman, 2010). It can also be applied together with experimental results to investigate the mechanisms behind a phenomenon of interest. Among these approaches, Monte Carlo and molecular dynamics simulations can describe particles by pairwise interaction potentials.

Molecular dynamics (MD) is a numerical simulation technique to calculate the thermodynamic and transport properties of many-body systems. The temporal evolution of a set of interacting particles is accompanied by the integration of classical equations of motion. The temporal averages of the trajectories and their fluctuations can be correlated with the macroscopic properties of the studied system (Tuckerman, 2010). Another approach is called Monte Carlo (MC), which is a method that relies on randomly finding the lowest energy state. Simplistically, this is accomplished by changing the system by one move for each Monte Carlo step, calculating the system’s free energy, and accepting or not the move based on that (Frenkel & Smit, 2001). Thus, MC is a simulation method that seeks to reduce the energy of the system in a stochastic way, while MD uses integration algorithms to solve the equations of motion of each particle following the system’s dynamics.

Both MD and MC can be applied in understanding the adsorption of pollutants to NPs and studying the interaction mechanism between NPs and biomolecules, such as phospholipids, proteins, deoxyribonucleic acid (DNA), and ribonucleic acid (RNA). Knowing the mechanism involved in these interactions is crucial to predicting dangerous combinations and outlining action strategies that reduce negative impacts on the ecosystems.

Despite the potential of molecular simulation to describe the mechanism of interaction between molecules and predict system equilibrium and dynamic properties, it has only been used to analyze systems with NPs and MPs in recent years. Therefore, this review aims to examine how research is being conducted in this area, identify how nanoplastics are being characterized within the molecular dynamics methodology, and to identify and analyze gaps in knowledge. These discussions become important to point out the paths to be followed and the difficulties that need to be addressed more urgently, as well as to guide those who are starting to simulate NPs and MPs while providing an overview for those already working in the area. This review focuses on four points: (i) summarize the force fields conventionally used to describe NP by molecular simulations; (ii) discuss the effects of NPs in the structural and dynamical properties of biological membranes; (iii) evaluate how nanoplastics affect the folding of proteins; (iv) and discuss mechanisms by which NPs and MPs adsorb contaminants from the environment.

## Survey Methodology

The combinations of keywords used in this research were: “nanoplastics molecular dynamics”, “microplastics molecular dynamics”, “nanoplastics Monte Carlo”, and “microplastics Monte Carlo”. To guarantee a thorough assessment of the literature, the keywords were searched in five different repositories: acs.org, Science.gov, sciencedirect.com, scopus.com, webofknowledge.com, and scienceresearch.com. In total, 706 results were found. Most of the articles were present in more than one of the repositories, thus guaranteeing an unbiased search. After analyzing the documents and excluding duplicates, 28 articles were considered with data that could be used in this review - basically, those that used molecular dynamics and/or Monte Carlo to study nanoplastics. It is interesting to highlight that, due to computational limitations, NPs are generally simulated rather than MPs. However, in many cases, the observations can be extrapolated to systems containing MPs. Meanwhile, there are situations in which the focus of interest is the NP itself.

## General remarks of the simulations and force field analysis

The number of scientific articles using the molecular dynamics approach has been growing since 2011, with five published papers from 2011 to 2017 and 23 published papers from 2018 to 2022. Only one paper briefly reported Monte Carlo simulations. Before discussing each point proposed in the study, it is necessary to comment on how research is being conducted in this area, and on important differences between said studies. Initially, it is possible to identify two groups of studies, the first focused exclusively on molecular dynamics, and the second focused on molecular dynamics as a complement to an experimental study or with other models. Within the first group, one can see the time scale used is mainly in the nanosecond range, reaching the microsecond range when using coarse-grained force fields (Bochicchio et al., 2017; Rossi, Barnoud & Monticelli, 2014). Within the second group, there are studies that simulate systems at the nanosecond scale, but also other studies that simulate at the picosecond scale, the latter mostly following an experimental study (Chen et al., 2021; Li et al., 2021b; Guo, Liu & Wang, 2020; Guo, Liu & Wang, 2019; Zhang, Zhao & Na, 2020; Qin et al., 2022). Considering that all studies work with the same type of system, *i.e*., a simulation box containing NP, a disparity between simulation times is noted, making a discussion about it worth having. This concern applies not only to studies of NPs through molecular dynamics, but also to any study that use this methodology. Braun et al. (2019), in their practice guide for molecular dynamics methodology, highlight that for condensed systems, depending on the type of information desired, there may be a dependence of the properties with the fluctuations and correlations of movement between the molecules. It is even mentioned that systems with polymers and proteins have relevant scales in the range of nanoseconds to microseconds, depending on what information one wants to obtain.

As mentioned previously, Monte Carlo and molecular dynamics can describe particles by pairwise interaction potentials. These interaction potentials are associated with the positions of the particles but also with parameters related to the substance, the particle size, bonds, dihedrals, among others. This information is computed into force fields that can be defined as the functional forms used to describe the intramolecular and intermolecular potential energies of the system. Many are the force fields available in the literature and their modifications, such as OPLS-AA (Optimized Potentials for Liquid Simulations, All-Atom) (Jorgensen, Maxwell & Tirado-Rives, 1996; Jorgensen & Tirado-Rives, 1988); AMBER (Assisted Model Building and Energy Refinement) (Ponder & Case, 2003); CHARMM (Chemistry at Harvard Macromolecular Mechanics) (Brooks et al., 2009); GAFF (General AMBER Force Field) (Wang et al., 2004); and TraPPE (Transferable Potentials for Phase Equilibria) (Martin & Siepmann, 1998). They were developed based on quantum mechanical calculations or experimental data. When working with molecular simulation, one of the main concerns must be the selection of the force field. In this section, we discuss the main force fields used in the parameterization of nanoplastics, the software used, and the chosen water models.

We noticed that five studies used some version of the Material Studio program (BIOVIA Dassault Systèmes, 2021) with the COMPASS force field (Sun, 1998), which is already available in the simulator program. Among all the software mentioned, this is the only one that is paid and has a friendlier interface; however, the software used must not interfere with the result. These studies have both MD and experimental sections, and deal with the sorption of contaminants on NP. All the experiments, but one, use between 100 and 500 ps of simulation time before analyzing the results. As already mentioned, relevant time scales for these systems should be higher in magnitude. An interesting fact is that none of them considered the presence of water, even if implicit, neglecting the fact that the phenomenon of interest occurs in an aqueous medium.

The most used molecular dynamics simulator to study NP was Gromacs (Lindahl et al., 2021), with 13 studies using its versions. The force fields were more varied in this case, but seven of those studies chose to use the GROMOS (GROningen MOlecular Simulation) (Scott et al., 1999) in one of its versions, which is the force field from the same developer as for the Gromacs. Five investigations used the LAMMPS simulator (Large-scale Atomic/Molecular Massively Parallel Simulator) (Plimpton, 1995), while one used the Gabedit program (along with the AMBER force field) (Allouche, 2011) and another used the Amber molecular dynamic package (Case et al., 2005). Of the works that used LAMMPS, two used OPLS-AA (Optimized Potentials for Liquid Simulations—All Atom) with SPC/E (extended simple point charge model) water model (Berendsen, Grigera & Straatsma, 1987).

Regarding the force field only, two publications combined GAFF (Wang et al., 2004) and TIP3P water model (MacKerell et al., 1998). One publication used GAFF (Wang et al., 2004) for both NP and water-the studies that used GAFF obtained the partial charges from different methods of quantum calculations. One publication combined TIP3P to water and a version of GROMOS to the NP. Three publications used OPLS-UA (Jorgensen & Tirado-Rives, 1988).

A force field development was carried out for polystyrene and polypropylene. It was specially designed for the interaction between these plastics with lipids. In other words, this force field (MARTINI coarse-grained (CG)) (Panizon et al., 2015) is compatible with the popular MARTINI force field for lipids (Marrink et al., 2007), and it was used in four different publications for experiments with simulation time from at least 100 to 20,000 ns. Tables 1 and 2 summarize the number of published studies on nanoplastic simulations *via* molecular dynamics and the force fields used to model NP and water in each of them. Hollóczki & Gehrke (2020) used the Automated Topology Builder and Repository (ATB) version 3.0, a website that provides classical molecular force fields for novel compounds (Malde et al., 2011). This investigation of Hollóczki & Gehrke (2020) together with the studies from Table 1 are the 28 referred articles in this literature review.

**Table 1:** Scientific publications applied to simulate nanoplastic *via* molecular dynamics or Monte Carlo and the force fields used to model the NP in each of them, identified by authors.

Force field	Number of articles	References
AMBER	1	Cortés-Arriagada (2021)
Compass	5	Chen et al. (2021), Li et al. (2021b), Guo, Liu & Wang (2019), Guo, Liu & Wang (2020), Yang et al. (2021)
GAFF	3	Wang et al. (2019b), Sarcletti et al. (2021), Ramalho, Dordio & Carvalho (2022)
GROMOS	7	Feng et al. (2022), Xue et al. (2011), Tan et al. (2020), Li et al. (2021a), Qin et al. (2022), Zhang, Zhao & Na (2020), Lameh, Baseer & Ashiru (2022)
MARTINI	5	Dettmann, Kühn & Ahmed (2021), Bochicchio et al. (2017), Li et al. (2021c), Panizon et al. (2015), Rossi & Monticelli (2014)
OPLS-AA	3	Hollóczki & Gehrke (2019), Hollóczki (2021), Rossi & Monticelli (2014)
OPLS-UA	3	Bochicchio et al. (2017), Bochicchio et al. (2022), Panizon et al. (2015)

**Table 2:** Scientific publications applied to simulate nanoplastic *via* molecular dynamics or Monte Carlo and the force fields used to model the water in each of them, identified by authors.

Water model	Number of articles	References
GAFF	1	Sarcletti et al. (2021)
MARTINI	1	Li et al. (2021c)
SPC	1	Xue et al. (2011)
SPC/E	2	Hollóczki & Gehrke (2019), Hollóczki (2021)
TIP3P	3	Wang et al. (2019b), Ramalho, Dordio & Carvalho (2022), Qin et al. (2022)
TIP4P	1	Toh et al. (2020)

## The effects of nanoplastics in the structural and dynamic properties of biological membranes

Biological membranes are complex structures composed basically of proteins and lipids stabilized by dynamic cooperative non-covalent interactions (Bogdanov & Dowhan, 2021). They are permeable protective barrier of the cells involved in relevant functions, namely sensing, transport, adhesion, and recognition processes. They consist of a bilayer of lipid molecules, and have functions such as the control of substances (*e.g*., ions, nutrients, waste) into and/or out of cells, keeping toxic substances outside the cells as well as separating vital, and often incompatible processes inside the cells (Watson, 2015). Biological membranes are very complex and, in addition to the lipid layers that allow or prevent the diffusion of smaller molecules, some proteins form channels that can allow free diffusion into and out of the cell and channels that only allow specific passage of some compound (Nelson & Cox, 2021). The imbalance of these processes may be linked to diseases such as cancer, neurodegeneration, and muscular dystrophies (Dias & Nylandsted, 2021). The manipulation of membrane dynamics has also been associated with anesthetic effects (Fábián et al., 2015). Thus, understanding, at the molecular level, how the interaction mechanisms of membranes with the environment works can influence our comprehension in more than one field of knowledge. In this section, we discuss recent advances in studies to understand how lipid membranes behave in the presence of NP contaminants through molecular simulations techniques.

The simulations made by Hollóczki & Gehrke (2020) show that a membrane composed by 2 × 300 molecules of 1-palmitoyl-2-oleoyl-phosphatidylcholine (POPC) (*i.e*., phospholipid commonly found in cell membranes) readjusts itself in the presence of nanoparticles of polyethylene with about 5 nm in diameter. The membrane rearranges to cover more of the NP, and the NP surface can rearrange itself to almost double in size in the presence of the membrane. The conformational changes gradually cause the membrane thickness to increase and the average area of each lipid to decrease during the 200 ns course of simulation time. In addition to structural changes, the results show that there are changes in dynamics as well since the presence of NP facilitates lipid movement within the membrane. Thus, it is suggested that the presence of NP has a significant effect on biological membranes (Hollóczki & Gehrke, 2020).

**Figure 2:**
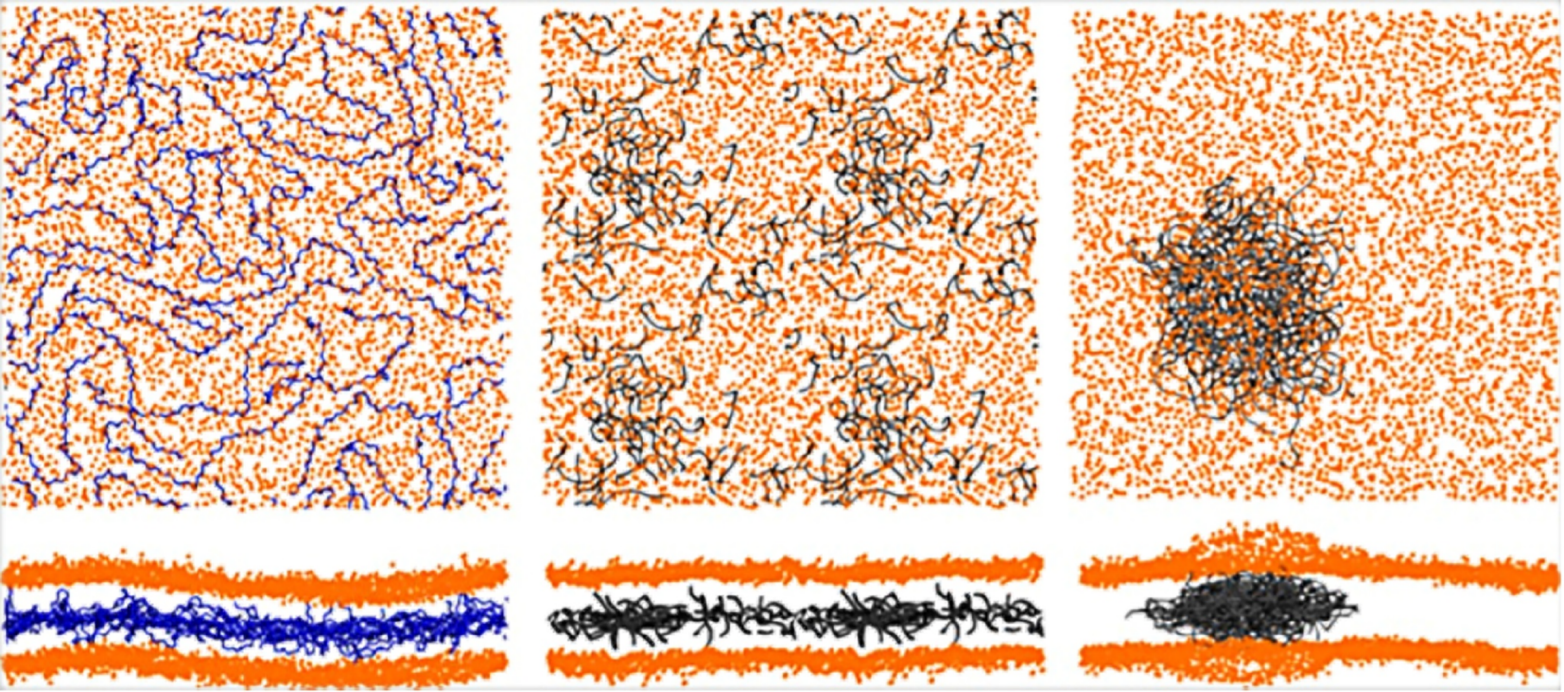
Typical distributions of the polymers inside pure POPC membranes (lipid:polymer mass ratio of 6.6%). Two views of the membrane (only head beads, in orange) are shown for each configuration: from the top and from the side. Left: long PP chains (in blue). Middle: short PE chains (in gray). Right: long PE chains (in gray). Reproduced from Bochicchio et al. (2017) - License CC BY 4.0 (http://creativecommons.org/licenses/by/4.0/).

Bochicchio et al. (2017) conducted a study using coarse-grained simulations of polyethylene (PE), polystyrene (PS), and polypropylene (PP) with diameters around 7 nm interacting with lipid membranes of POPC. All NPs quickly entered the membrane and changed their behavior from solid to liquid at room temperature. Depending on the type of NPs and on their degrees of polymerization, one can observe different situations: polyethylene chains tend to aggregate when in a high polymerization degree, unlike the other two NPs whose chains tend to separate from each other, as shown in Fig. 2. An interesting topic of this work is the study of heterogeneous membrane systems (those composed of ternary lipid mixture exhibiting liquid-ordered/liquid-disordered phase separation). These membranes are formed by more than one type of lipid, so it is possible to observe the dynamics of a more complex system. Once again, the result was related to the type of polymer: the separation of the lipid phase is disadvantaged by PP, while PS stabilizes the lipid phase, and PE modifies the boundary topology and causes cholesterol depletion from the liquid-ordered phase. The authors emphasize the need for further studies to better understand the toxicity of NPs that humans have come more and more in contact with recently. This study follows up a previous one (Rossi, Barnoud & Monticelli, 2014) which is simpler in the variety of compounds and deals with different sizes of sets of polystyrene chains and a POPC membrane. Initially, they carried out an extensive study of the coarse-grained force field and, only after good agreement with the OPLS-UA force field, they performed the simulations with PS chains formed by 10, 20, and 100 monomers. In equilibrium, the set of shorter chains (with less than 100 monomers) were dispersed in the membrane, not aggregating, whereas the one with 100 monomers preferred to be concentrated in the center of the membrane. The results show that PS alters the properties of the membrane, significantly increasing its compressibility modulus and decreasing its bending modulus, which indicates a structural change in the membrane in addition to affecting its lateral organization.

In the wake of these last two investigations, a recent publication presented experimental and molecular dynamics results, introducing small chains (with 25 styrene monomers) in dipalmitoyl-phosphatidylcholine (DPPC) lipid bilayers (Bochicchio et al., 2022). The authors carried out the equilibration for 50 ns of three systems composed of the DPPC membrane and different mass ratios of PS. Because they use the OPLS-UA force field (*i.e*., coarse grained-force field), it was possible to simulate up to 2 microseconds in the production stage. The experimental and molecular dynamics results complement each other and lead the authors to believe there is a critical concentration in partial segregation of PS chains within the membrane. Similarly to Hollóczki & Gehrke (2020), Bochicchio et al. (2022) showed changes in membrane thickness. The authors reported a nonlinear increase in diffusion coefficients with the PS mass ratio (Bochicchio et al., 2022). For the bending modulus values, the behavior was inverse in the presence of PS. There was a substantial decrease in bending modulus compared to the total absence of PS. The results confirm that the effects of NPs on human health cannot be underestimated, and that concentration is a factor to be analyzed.

The last study to be covered by this section brings an exciting view of how treating water with chlorine can affect the MPs to the point of increasing the toxicity of these MPs (Qin et al., 2022). The authors conducted experiments, and, using MD in accordance to the methodology of Bochicchio et al. (2022), they compared the system of pristine polystyrene MPs and chlorinated pristine polystyrene MPs (with chlorine- and oxygen-containing functional groups) in contact with bilayer membranes of phosphatidylcholine. Simulations showed that MPs interact differently with membranes and the authors speculate that the increased cell membrane permeability caused by chlorinated PS might be due to the presence of C-Cl bonds. Consequently, the presence of chlorinated pristine polystyrene MPs can cause even more damage at the cellular level (Qin et al., 2022).

All studies showed changes in lipid membranes when in contact with NPs or MPs. Considering the biological functions of membranes, it is plausible to conclude that these NPs/MPs somehow affect the cellular environment. All investigations suggested that further investments in this field are needed for proper assessment and control of the potential negative effects of NPs/MPs on environment and human health.

## Nanoplastics affecting the folding of proteins

Proteins play a substantial role in human health. They are critical for tissue growth and maintenance (Yeung et al., 2017), and for various biochemical reactions when proteins take the form of enzymes, with functions such as digestion, blood clotting, energy production, and muscle contraction (Hatzimanikatis et al., 2004). Equally important, proteins can also act as hormones that are chemical messengers responsible by the communication between cells, tissues, and organs (Nussey & Whitehead, 2001). Additionally, they are responsible for regulating the pH of body fluids (Hamm, Nakhoul & Hering-Smith, 2015), such as blood and stomach acid, and acting in the body’s defense (Li et al., 2007). In recent years, studies that associate the presence of NPs in the human body with changes in protein structures began to emerge. The molecular dynamics methodology is currently used to understand the dimension of these changes and their possible effects on human health. It is proven that NPs tend to interact with proteins to the point of modifying their secondary structures, with the result being protein denaturation (Hollóczki & Gehrke, 2019).

**Figure 3:**
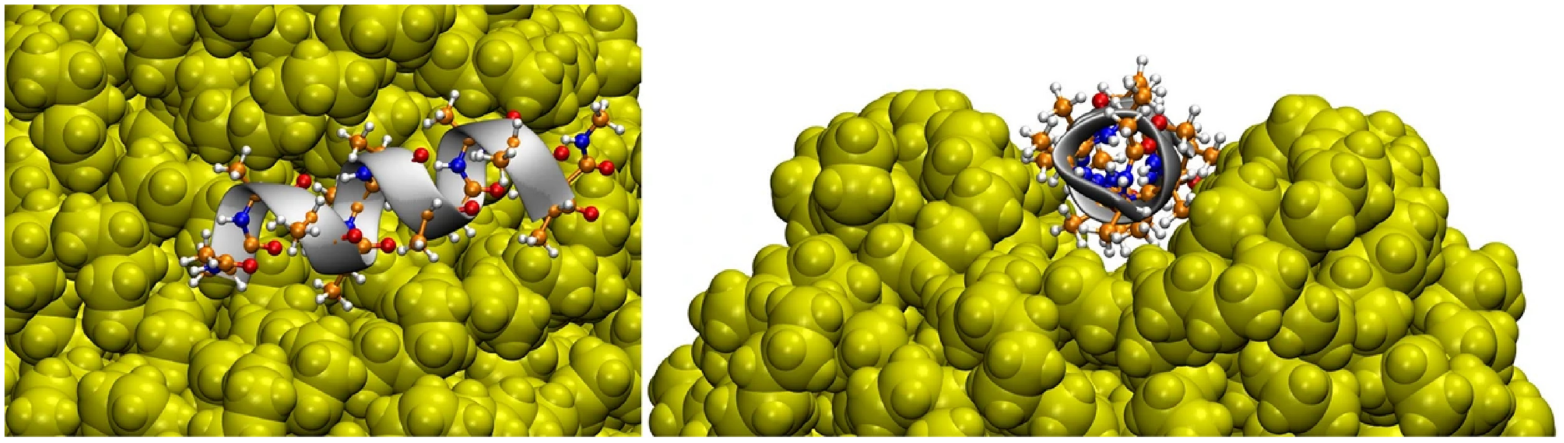
Three-dimensional structure of the helical peptide (composed of 12 alanine units) on thesurface of a PE nanoparticle (yellow) from two views. Reproduced from Hollóczki & Gehrke (2019) - License CC BY 4.0 (http://creativecommons.org/licenses/by/4.0/).

Hollóczki & Gehrke (2019) studied four types of NPs interacting with a series of proteins, namely PE, PP, nylon-6,6, and polyethylene terephthalate (PET). They showed, for instance, that the amino acids polarity is a relevant factor in their adsorption on NPs. Non-polar amino acids such as phenylalanine and tryptophan tend to have such a high interaction with NPs that basically all amino acids in solution adsorb to NPs. They can form a micelle around NP, showing that the hydrophobicity of NPs can be masked by proteins, affecting their solubility and ability to aggregate (Hollóczki & Gehrke, 2019). It is interesting to highlight that the interaction between NPs and tryptophan zipper induced no significant changes in its overall structure, regardless of the adsorption of the peptide on the surface of the NP. However, the lack of structural rearrangement at the end of the simulation does not mean that the NP does not affect the protein structure because the time scale for this to happen could be greater than that available for the simulation. Moreover, a *α*-helix composed of 12 alanine adsorbed on hydrophobic surfaces of NP, and mainly for PE and PP, the nanoparticle rearranged to incorporate the peptide, affecting the conformation of the protein, as shown in Fig. 3. Another relevant point is that the results differ greatly depending on the NP, *i.e*., the problems caused by exposure to NPs are different depending on their type. This was the first article that had Hollóczi, notably the author of the most publications regarding the study of NPs with molecular simulations, published in said field.

Later, Hollóczki (2021) single-handedly published an article on the same topic. The study used, in addition to MD, the quantum chemistry theory. In order to overcome the limitations of MD regarding the time scale and the energetic barriers of the restructuring of compounds as proteins, which were pointed out even in the previous work (Hollóczki & Gehrke, 2019), Hollóczki (2021) discussed the use of simulated annealing (SA) strategy. In this methodology, one runs a simulation with the system at high temperatures allowing several conformations to be accessed, and then, the system is gradually cooled to, ideally, the minimum free energy level. Hollóczki (2021) used this methodology to find the best conformations for polyethylene and nylon-6,6, reaching the conclusion that the temperature range for carrying out the SA must be approximately between the condensation and freezing temperatures of the compound. This methodology proved to be quite efficient in revealing important structural information. Peptides’ structures were, thus, optimized through quantum chemistry and then submitted to SA. The next step was to run SA for the peptide-nanoplastic pair. About 150 simulations were performed for each one of them. Further optimizations were performed using MD and quantum chemistry at the end of the simulations, and then the adsorption, interaction, and reorganization energies were defined and calculated. In this study, the two plastics influenced the stability of the secondary structure of the simulated peptide, corroborating previous results. The need for further studies to understand the consequences of these changes is also highlighted.

In a more recent study, Li et al. (2021a) performed an experimental investigation coupled with a simulation study of insulin fibrillation in the presence of nanoplastics and various contaminants. They chose insulin to represent the protein, because it is a model already widely used in the literature in the study of protein fibrillation and conformational changes. The research aimed to examine whether common organic contaminants (pyrene, bisphenol A (BPA), 2,2, 4,4-tetrabromodiphenyl ether (BDE47), 4,4-dihydroxydiphenylmethane (BPF), and 4-nonylphenol (4-NP)) enhance the abilities of NPs to accelerate insulin fibrillation as well as carry out a molecular study of the mechanism of action. By MD, the insulin, pyrene insulin and polystyrene insulin systems were studied. Although one of the goals was to examine the three dynamics together, the simulations were done in pairs. The presence of polystyrene had a more significant effect on the conformational transition of insulin than that which was observed with the presence of pyrene, which is consistent with the trends in the experimental results. It is possible to conclude that van der Waals forces predominate in insulin binding with PS or pyrene. These results complemented previous studies of the mechanism by which NPs promote insulin fibrillation.

Another study involving NPs and proteins deal with the effect of using PEG spacer on small peptides in a process commonly called PEGylation (Xue et al., 2011). This process is used to stabilize, immobilize or modify biomolecule properties. The authors sought to understand, at the molecular level, the structural effects caused on the peptides due to the PEGylation. The peptides were simulated under three conditions: free in solution, attached to a PEG spacer, and attached to PEG spacer constrained to a two-dimensional lattice to mimic the display of a peptide on the surface of a microsphere. The results suggested that the no charged peptides in a PEGylation situation do not undergo a noticeable conformational change. However, peptides with high charges, both negative and positive, suffered conformational changes. These results show the need for specific studies and significant investment in this research field. Since we were unable to find additional reports focusing on this specific subject, it was not possible to compare results. This fact alone justifies the relevance of more investment to further develop the field. The mentioned study is presented here distinctly from the other works in this section due to its author discussing a process in which peptides are artificially changed through a PEG spacer while acting as a single unit, instead of examining the interaction between the protein and the microplastics separately.

In general, the study of interactions between NPs and proteins shows that a single type of nanoplastic cannot be used to understand the possible effects of contacting all types of nanoplastics with proteins. More than one of these studies discussed that polarity is fundamental in how NPs and proteins interact. It is also possible to conclude that this type of study is necessary to indicate new research routes in the subject. Different pairs, peptides, and NPs should be analyzed to understand the problem to a greater. The present number of investigations in this area is still insufficient to provide a general dimension of what must be approached.

## Sorption of contaminants on nanoplastics

Research has been conducted to investigate the effects of microplastic as a vector for POP (persistent organic pollutants) contamination in the aquatic environment (Bhagat, Nishimura & Shimada, 2021; Rodrigues et al., 2019; Koelmans et al., 2016). MPs and NPs present physicochemical properties, such as surface area, shape, chemical composition, functional groups, and surface charge, that promote their interactions with different organic and inorganic pollutants in the environment (Zhang et al., 2020; González-Pleiter et al., 2021; Bhagat, Nishimura & Shimada, 2021; Wright, Thompson & Galloway, 2013). Although some studies emphasize that the ingestion of microplastic contaminated with POP does not increase the risk for the marine animals when compared to the flux of POPs bioaccumulated from natural prey (Koelmans et al., 2016), others have focused on the potential harm of this combination, highlighting a possible existence of synergistic effects between MPs and POPs (Bhagat, Nishimura & Shimada, 2021; Rodrigues et al., 2019). However, it remains unclear whether this relationship actually exists under environmentally relevant conditions. More studies are needed to determine the actual capacity of NPs/MPs to transport associated pollutants that result in trophic transfer and bioaccumulation in the food chain. Experimental studies should be more widely developed based on actual conditions, mainly, concentration and nature of both MPs/NPs and contaminants commonly found in aquatic environments, combined with ecotoxicity assays with organisms of different trophic levels. While the experimental development continues to grow, simulation studies help indicate the paths to follow. In this field, in terms of MPs, the most studied polymer types consist of PE, PP, PS, polyamide (PA), and polyvinyl chloride (PVC) (Bakir, Rowland & Thompson, 2012; Fries et al., 2013).

Organochlorine pesticides (OCP), polychlorinated biphenyls (PCB), polybrominated diphenyl ethers (PBDE), and PAH are some chemicals that can accumulate on MPs (Bakir, Rowland & Thompson, 2012; Fries et al., 2013; Rodrigues et al., 2019). In addition, MPs and NPs can release chemical monomers and additives with proven toxicity that are incorporated into materials during their manufacture, such as plasticizers, flame retardants, antimicrobial agents, and pigments (Fries et al., 2013). The sorption depends on the system properties (*e.g*., temperature and pH) and the physical-chemical characteristics of the polymer and the contaminant.

The main sorption mechanisms between chemical compounds and MPs/NPs are hydrophobic interaction, electrostatic interaction, pore filling, van der Waals forces, hydrogen bonding, and ${\pi - \pi}$ interaction (Tourinho et al., 2019). Experimental investigations can provide evidence of the most likely type and mechanism of interaction; however, computer simulations are a valuable tool to predict and understand these processes. Moreover, the simulations can guide the experimental assays to look to the compounds with chemical characteristics that are harmful or interact more with a specific polymer.

Guo, Liu & Wang (2019) analyzed the sorption of sulfamethazine on PE, PS, PP, PA, PET, and PVC. To build the simulation box, only one polymer chain and one molecule of sulfamethazine were added in the vacuum for each system. The number of monomers varies from 100 to 300 depending on the polymer type. Besides, the simulation was performed in NVT ensemble at 298 K for 500 ps. The results suggested a dominance of electrostatic interactions in the sorption on PA, PS, PVC, and PP. In contrast, van der Waals interactions were dominant in the sorption on PE and PET. The computed adsorption energies decreased in the order PA > PET > PE > PVC > PS > PP.

Another antibiotic that was investigated was the tetracycline hydrochloride and its derivatives (chlortetracycline hydrochloride and oxytetracycline hydrochloride) (Chen et al., 2021). The simulation box consists of a PE chain with 300 monomers degree of polymerization, and one antibiotic molecule. The simulation was performed in NVT ensemble at 298 K. The interaction energy reveals that the adsorption capacity of tetracycline hydrochloride on PE is the weakest, whereas the chlortetracycline hydrochloride presents the highest adsorption capacity. Chen et al. (2021) also analyzed the radial distribution functions, which indicated a preference for the non-bond interaction between the carbon atoms of PE and the oxygens in the tetracycline molecules.

The adsorption of three pesticides (difenoconazole, buprofezin, and imidacloprid) on the PE molecular chain with polymerization degree equal to 160 was also evaluated, and interaction energies were found to be statistically the same among them (Li et al., 2021b). Besides, the MD simulation used the Grand Canonical Monte Carlo method without giving more details about the properties analyzed with this method (Li et al., 2021b).

Regarding PAH, Yang et al. (2021) analyzed the sorption of pyrene, 1-methylpyrene, 1-hydroxypyrene, 1-aminopyrene, and 1-pyrenecarboxylic acid on PS with polymerization degree equal to 100, and found that said interaction had higher levels of energy when compared to sulfamethazine (Guo, Liu & Wang, 2019); however, the values for pyrene and its derivative were very close one another. Following the experimental evidence and the computational results, the pore-filling and the hydrophobic and ${\pi - \pi}$ interactions played an essential role in these adsorptions (Yang et al., 2021).

The sorption of SrCl_2_ on PA, PS, and PP was also investigated, and the electrostatic interactions were the dominant mechanism (Guo, Liu & Wang, 2020). The simulation box was built following the methodology described by Guo, Liu & Wang (2019). For SrCl_2_, the adsorption energies followed the order PP > PS > PA, smaller than NPs and organic pollutants.

It is important to highlight that the simulations reported by Guo, Liu & Wang, 2019, Guo, Liu & Wang (2020), Li et al. (2021b), Chen et al. (2021), and Yang et al. (2021) were conducted in the absence of water, even if implicit. The simulation boxes were composed of one polymer chain, one molecule of the pollutant and a vacuum layer. Although this kind of simulation requires less computational time, the validation is questionable when compared to experimental results in an aqueous medium, since the simulations were conducted in a vacuum.

Cortés-Arriagada (2021) studied the co-transport of BPA with PET using density functional theory (DFT) with B3LYP functional at def2-SVP basis sets. The solvent was considered implicitly, *i.e*., a continuum medium characterized by the dielectric constant. To obtain an initial configuration of the PET nanoplastic, MD was performed by folding a single polymer chain described by AMBER force field using NVT ensemble, followed by energy minimization steps. The final nanoPET model was optimized *a posteriori* at the DFT level. Due to the nucleophilic nature of the outer surface of nanoPET and the hydrophobic characteristic of BPA, mass transfer and intraparticle diffusion of the pollutant into the nanoplastic were observed. An interplay between dispersion and electrostatics intermolecular interactions occurred, with the former dominating the inner surface adsorption, whereas the latter dominated the outer surface adsorption.

Feng et al. (2022) aimed to understand the process of aggregation of humic acid molecules (HA) with the contaminant benzo[a]pyrene (BaP) and heavy metal ions (Cu^2+^) in an aqueous solution, as well as the influence of PS, PP, PVC, PET, and PE in these systems. Simulations with NP, HA, BaP, and Cu^2 +^ show a competition between HA and BaP to adsorb on NP. When HA wins the competition and adsorbs on NP, it exposes carboxyl groups that offer interesting binding sites for Cu^2 +^ adsorption. The results indicate that PS has the highest capacity of adsorbing BaPs. The motivation to study this system comes from the fact that environmental factors (such as dissolved organic matter - in the article represented by HA) can influence the interactions of NPs and contaminants. Hence the necessity to consider these factors in more detailed studies.

There are infinite combinations of pollutants and NPs that humans can contact, and each one can result in different consequences. However, the more studies in this field, the more it is possible to extrapolate behavioral trends. Thus, the investment of resources in this area becomes essential.

## Studies that used molecular simulation and do not fit into the above sections

Although most MP/NP studies *via* molecular simulation can be fitted into the sections above, some address more specific issues. Zhang, Zhao & Na (2020) conducted combined research with molecular dynamics, ratio normalization, and molecular docking methods to understand biodegradation of phthalic acid esters (PAE) derivatives in marine and fresh-water environments. PAE are commonly present in plastics and confers their characteristics of malleability and plasticity; thus, they were chosen to serve as a model for the study of the biodegradability of plastics in marine environments. As we have already discussed, marine environment ends up being the final destination for tons of plastics every year, which is why the study is so important. As a result, five PAE derivatives were designed with excellent biodegradability as a goal, as well as functionality in mind.

Following the same train of thought, molecular dynamics and molecular docking appeared in Lameh, Baseer & Ashiru (2022) study about mutating existing enzymes to facilitate the biodegradation of PET and PP. The authors modified certain amino acids of the enzyme *Archaeoglobus fulgidus* (AFEST) and compared the changes in binding energy with plastics. They claimed that modifying an existing enzyme is the best approach, from the biotechnology viewpoint, to solve our plastic waste problem.

Moreover, Wang et al. (2019b) shared some exciting findings using molecular mechanics Poisson-Boltzmann surface area method and molecular dynamics simulations. Through prior knowledge that the hydrolase enzyme RPA1511 (obtainable from *Rhodopseudomonas palustris* bacterium) can efficiently depolymerize polylactic acid (PLA) plastic, they sought to understand which amino acids are responsible for this action. The binding affinity data showed that the RPA1511 could also degrade other polyester plastics. These results open doors for the study of more biodegradable plastics.

Another interesting research was that of Ramalho, Dordio & Carvalho (2022). They carried out extensive molecular dynamic simulations of PE, nylon 6 and PET in an aqueous environment to achieve compression, mainly from their conformational behavior, of polymer chains after exposure to water. Over 200 ns of simulation, the three plastics had different responses, and, in the end, their chains equilibrated in the following ways: compacted and ordered, almost like a crystal, for PE, globular chains for nylon 6, and, for PET, tangled chains with the aromatic rings preferably oriented in parallel. Understanding how these plastics organize themselves when they are in such small particle form demonstrates how other contaminants can adsorb onto their surfaces and cause even more damage.

Since the presence of NPs in water is a reality, and, with research showing their potentially harmful effects, a line of study arises naturally: the search for methodologies for the removal of NPs from aqueous environments. Sarcletti et al. (2021) came up with the idea of removing NPs from water by applying an external magnetic field. They developed superparamagnetic iron oxide nanoparticles (SPION) that attract NP; they worked with both structural analysis, and molecular dynamics which supported the experimental results.

As far as we could track, the work by Dettmann, Kühn & Ahmed (2021) is the only one that applied MD to NPs found in soils. The authors used carbon nanotubes (with and without functional grouping) as a model for hydrophobic cavities and surfaces to represent an existing structure in organic soil particles. They carried out up to 500 ns of coarse-grained molecular dynamics simulations of hydrophilic and hydrophobic NP, carbon nanotubes, and water. NPs behave as expected concerning the hydrophobic carbon nanotubes according to their hydrophobicity. Regardless, the study stand out for being the first step in understanding processes in environments such as soils.

The work by Li et al. (2021c) about the impact of NP inhalation on the lungs is extremely interesting and pertinent. They investigated five types of NPs varying their sizes, surface charges, and molecular weights, and exposed them to interaction with lung surfactant (LS) film, both in the alveolar fluid and at the air-water interface. The authors pointed out that although the study does not yet represent an authentic environment in its complexity, the type of NP, its size, surface charge, and molecular weight were factors that modified the results in the interaction with LS. A study along the same lines (except that, rather than the concern about inhaling MPs and NPs, it deals with impacts on the human intestinal tract) is that of Tan et al. (2020), which shows results on how five different types of MPs reduce lipid digestion when ingested with food. This work presents molecular dynamics and experimental results, with the former corroborating the latter’s findings and going further in understanding interactions between NPs and lipids. The authors conclude that the interaction between lipid droplets and MP is expected to play an influential role in reducing lipid digestion.

Although we initially sectioned this review to manage the study in a more organized way, several other kinds of research were found that do not fit into any of our sections. We can highlight the studies that follow the line of seeking alternatives for removing NPs and MPs from the environment with which humans come into contact, mainly water. It is clear there are numerous ways to approach the problem of NPs and MPs.

## Conclusions

The present literature review focus on molecular simulation methodologies to study MPs and NPs interactions with proteins, biological membranes, and other contaminants, the force fields used, and the main findings.

Most scientific publications are very recent, which strongly indicates that the subject is growing in importance. That is mainly because MPs/NPs released in very high quantities by human activities end up, mostly, in aquatic and marine environments. However, the interactions with and potential impacts on living organisms are largely unknown. All studies regarding the consequences of human contact with MPs/NPs have been hypothetical, while showing that MPs/NPs interact with their surroundings, fundamentally modifying their characteristics.

The MD simulation was the most used model out of the methodologies applied, and, based on the results, it fulfilled its objective in showing the interactions at the molecular level. An obstacle, however, is the level of simplification that is necessary during simulation, since natural systems, given their concrete complexities are still outside the reality of investigations on a molecular level. Despite this, simulations can help a great deal to understand experimental data. The use of both experimental and computational approaches is in many scientific reports, and, in their conclusions, the authors have pointed out that they complement each other. As this is a fairly new field of study, there is no good methodology protocol to date on how to simulate NPs. Therefore, the steps in the methodological approaches vary considerably from study to study. Among these differences, one can highlight the simulation time, the force fields applied, the presence or absence of water models, and how the polymer chains are built to be considered NP particles. It is necessary to discuss the validity of certain practices within the molecular simulation to create a more mature protocol based on the information accumulated. In addition to MD, another promising option is the MC methodology. Although many publications presented equilibrium properties that can be accessed through both MC and MD, only one investigation reported the use of MC methodology. Due to how the system reaches equilibrium, MC could be an alternative to achieve simulations with shorter computational times. Between the results obtained, it is interesting to highlight that the interactions between all NPs and the environment cannot be understood through a single nanoplastic model. Depending on the NP type, the interactions, whether with proteins, lipids, or contaminants, are expected to differ significantly. Thus, each NP may cause a different impact when in contact with humans and other living organisms, which makes further studies even more pressing.

In future perspectives, it would be also interesting to investigate the effect of NPs in DNA and RNA, and to include the effects of plastic additives on the molecular interactions of MPs and NPs with contaminants and biomolecules.

## Additional Information and Declarations

### Data Availability

The following information was supplied regarding data availability: This is a literature review.
